# Prediction of Drug-Drug Interactions with Bupropion and Its Metabolites as CYP2D6 Inhibitors Using a Physiologically-Based Pharmacokinetic Model

**DOI:** 10.3390/pharmaceutics10010001

**Published:** 2017-12-21

**Authors:** Caifu Xue, Xunjie Zhang, Weimin Cai

**Affiliations:** Department of Clinical Pharmacy and Pharmaceutical Management, School of Pharmacy, Fudan University, 826 Zhangheng Road, Shanghai 201203, China; 15111030045@fudan.edu.cn (C.X.); 12307120290@fudan.edu.cn (X.Z.)

**Keywords:** physiologically based pharmacokinetic model, drug-drug interactions, bupropion, hydroxybupropion, threohydrobupropion, erythrohydrobupropion, inhibitory metabolites

## Abstract

The potential of inhibitory metabolites of perpetrator drugs to contribute to drug-drug interactions (DDIs) is uncommon and underestimated. However, the occurrence of unexpected DDI suggests the potential contribution of metabolites to the observed DDI. The aim of this study was to develop a physiologically-based pharmacokinetic (PBPK) model for bupropion and its three primary metabolites—hydroxybupropion, threohydrobupropion and erythrohydrobupropion—based on a mixed “bottom-up” and “top-down” approach and to contribute to the understanding of the involvement and impact of inhibitory metabolites for DDIs observed in the clinic. PK profiles from clinical researches of different dosages were used to verify the bupropion model. Reasonable PK profiles of bupropion and its metabolites were captured in the PBPK model. Confidence in the DDI prediction involving bupropion and co-administered CYP2D6 substrates could be maximized. The predicted maximum concentration (C_max_) area under the concentration-time curve (AUC) values and C_max_ and AUC ratios were consistent with clinically observed data. The addition of the inhibitory metabolites into the PBPK model resulted in a more accurate prediction of DDIs (AUC and C_max_ ratio) than that which only considered parent drug (bupropion) P450 inhibition. The simulation suggests that bupropion and its metabolites contribute to the DDI between bupropion and CYP2D6 substrates. The inhibitory potency from strong to weak is hydroxybupropion, threohydrobupropion, erythrohydrobupropion, and bupropion, respectively. The present bupropion PBPK model can be useful for predicting inhibition from bupropion in other clinical studies. This study highlights the need for caution and dosage adjustment when combining bupropion with medications metabolized by CYP2D6. It also demonstrates the feasibility of applying the PBPK approach to predict the DDI potential of drugs undergoing complex metabolism, especially in the DDI involving inhibitory metabolites.

## 1. Introduction

Metabolized drug-drug interactions (mDDIs) have been one of the main reasons for the failure of new drug research and development; a variety of drugs have been forced to withdraw from the market due to serious DDIs [1,2,3,4]. With the increasing and development of new drugs and usage, clinical combination therapy has become very common and inevitably increases the probability of occurrence of DDI. Consequently, evaluation of a potential risk of mDDIs is essential to improve safety and minimize the clinical risks associated with drug interactions [5].

In general, metabolites are formed primarily via metabolic enzymes, which play an important role in pharmacological activity and toxicity. Compared to the parent drug, it is generally considered less likely to cause metabolized drug interactions due to more polarity. In vitro studies of parent drugs are sufficient to avoid DDI risks [6].

However, it has recently been found that some important metabolites of inhibitors also have inhibitory effects [6,7,8]. In the latest Food and Drug Administration (FDA) draft guidance [9], it is explicitly stated that metabolites should be studied in DDI if the metabolite’s area under the plasma concentration-time curve (AUC) is greater than or equal to 25% of the parent AUC (AUCm/AUCp ≥ 0.25). The European Medicines Agency (EMA) further emphasizes that, for metabolites with AUCm/AUCp > 0.25 and represent >10% of total drug-related material [10], it is recommended to evaluate their DDI. In addition, regulators are also strongly proposing to predict and understand potential clinical DDI from the perspective of physiologically-based pharmacokinetic (PBPK), especially those complex DDIs [9,10,11]. The PBPK model provides a dynamic method for evaluation of DDI based on the physiological mechanism [12,13,14,15]. Compared with the static approach, it is reasonable to anticipate that the dynamic model is more accurate in the predication of DDIs, such as simultaneous inhibition and induction [16,17], the DDI of both substrate and inhibitory metabolites [15,18,19] and multiple DDIs. Recently, PBPK models have been widely applied in research and development, and even some good models are accepted by regulatory agencies and can be used to exempt some clinical trials [11,20,21,22,23].

**Figure 1:**
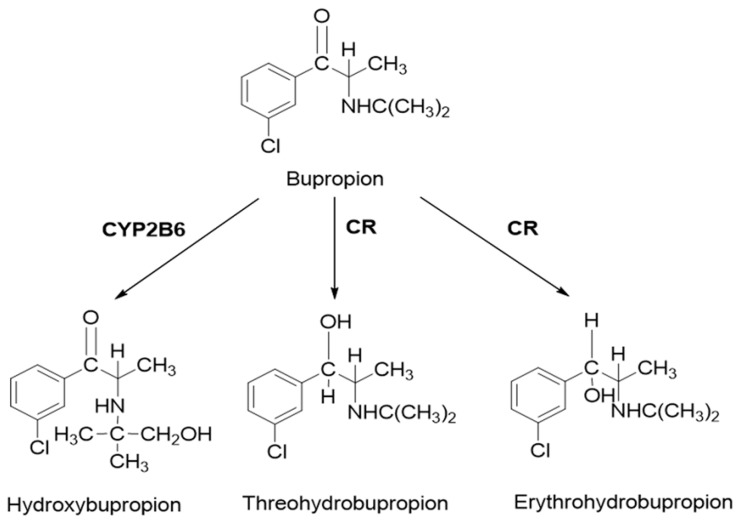
Bupropion and metabolism. Bupropion is metabolized by CYP2B6 to form hydroxybupropion and by carbonyl reductase to form the diastereoisomers threohydrobupropion and erythrohydrobupropion. CR: carbonyl reductase.

Bupropion is widely used in the treatment of major depressive disorder and smoking cessation. As a classical probe substrate for CYP2B6, it is metabolized to hydroxybupropion. In human, carbonyl reductase also plays an important role in the metabolism of bupropion. Threohydrobupropion and erythrohydrobupropion are two major metabolites produced by the reduction of the carbonyl group [24,25,26,27] (Figure 1). Although bupropion is not a substrate for CYP2D6, it also inhibits CYP2D6 activity [27,28]. Clinical studies have shown that there is a significant increase in substrate exposure when bupropion was administered in combination with substrates for CYP2D6. For desipramine, a five-fold increase in exposure was caused [29]. However, in vitro studies have shown that bupropion and hydroxybupropion are weak CYP2D6 inhibitors (IC50 = 58 and 74 μM, respectively) [27]. Thus, bupropion was chosen as the model drug. To better understand the complex DDI, a PBPK model was taken in the present study.

The objectives of the present work are (1) to build a PBPK model that can describe the PK profile of bupropion, hydroxybupropion, threohydrobupropion and erythrohydrobupropion; (2) to verify the bupropion PBPK model on the basis of the results of different-dose bupropion PK studies; and ultimately (3) to apply the PBPK model to predict the clinically observed DDIs with bupropion and its metabolites as the CYP2D6 inhibitors, and to better understand the involvement and impact of inhibitory metabolites for DDIs.

## 2. Materials and Methods

### 2.1. Physiologically-Based Pharmacokinetic (PBPK) Model Development

The Simcyp software package version 15 (Simcyp Limited, a Certara company, Sheffield, UK) was used to develop the PBPK model of bupropion and its metabolites. The absorption and distribution of bupropion was described by the first-order absoption and full PBPK model. For other metabolites, a minimal PBPK model were used to describe their distribution. To better predict the DDIs involving bupropion and its metabolites as CYP2D6 inhibitors, the model was first developed to simulate the PK of bupropion, hydroxybupropion, threohydrobupropion and erythrohydrobupropion when bupropion was given in different doses. Then, the verified model was used for the prediction of the involvement and impact of inhibitory metabolites in DDIs. Bufuralol, tolterodine, metoprolol, desipramine, and dextromethorphan as the CYP2D6 substrates from the Simcyp simulator library were used to simulate the DDIs. In addition, the PBPK model of venlafaxine was also built to simulate the DDI with bupropion. The observed clinical data were digitized from the graphs provided in literature using DigIt version 1.04 (Simulations Plus, Inc., Lancaster, CA, USA), a plot digitizer tool.

### 2.2. PBPK Model for Bupropion

The physicochemical properties of bupropion, including molecular weight, logP, pKa, blood-to-plasma ratio and fraction unbound in plasma were obtained from literature and in silico prediction as listed in Table 1. Bupropion binding to human plasma protein is 82% to 88%. Its absorption was described with a first-order absorption model. It has been reported that the absorption of bupropion is close to 100% [26]. A full PBPK model was used to describe the distribution of bupropion. The distribution of bupropion was predicted with Rogers method [30] based on the fitted K_p_ scalar to comparable to the observed value of 19 L/kg [31]. Bupropion is mainly metabolized by the liver and less than 1% of the parent drug is found in the urine [26,29]. According to the in vitro studies [27,32,33] with human liver microsomes, the enzyme kinetic parameters (V_max_ and K_m_) of bupropion to form hydroxybupropion, threohydrobupropion and erythrohydrobupropion were integrated into the model. Considering the other metabolic pathways of bupropion, the formation of threohydrobupropion and erythrohydrobupropion were by carbonyl reductase. Therefore, in this model, we assumed that threohydrobupropion and erythrohydrobupropion were cleared likewise by CYP2B6. The f_u,mic_ is used to correct the expression of carbonyl reductase to obtain the best simulation results closest to observed data.

### 2.3. PBPK Model for Hydroxybupropion, Threohydrobupropion and Erythrohydrobupropion

The physicochemical properties of the three metabolites were obtained from in silico prediction. The distribution of metabolite hydroxybupropion, threohydrobupropion and erythrohydrobupropion were described by a minimal-PBPK distribution model with tissue partition coefficients predicted by the Rodgers method [30]. A single adjusting compartment in Simcyp optimized the V_ss_ of hydroxybupropion and threohydrobupropion. The elimination of all metabolites are fitted based on the corresponding observed clinical data. The corresponding parameters are listed in Table 2.

### 2.4. PBPK Model for Venlafaxine

In addition, venlafaxine was also used as a CYP2D6 substrate to run the simulation with bupropion. To simulate the DDI between bupropion with venlafaxine, a PBPK model for venlafaxine was developed. The model of venlafaxine was built by a minimal PBPK model with tissue partition coefficients predicted by the Poulin and Theil method [35] combined with a first order absorption. The model parameters of venlafaxine are placed in Table 3. An oral absorption up to 92% was found. The K_p_ scalar of 2.3 was used to predict the V_ss_ comparable to the observed value of 7 L/kg [36,37,38]. The plasma protein binding of venlafaxine is low at 27% [37]. There is a consensus that the metabolic pathway of venlafaxine is mediated predominantly by CYP2D6. The CYP2C19, 2C9, and 3A4 isoforms also play a role in the metabolism of the drug, but to a lesser extent. The elimination of venlafaxine is fitted based on the corresponding observed clinical data. The intrinsic clearance (Clint) was calculated using retrograde model, assuming 80% Hep CL via CYP2D6 [39].

### 2.5. Simcyp Simulation

The Simcyp software package version 15 (Simcyp Limited, a Certara company, Sheffield, UK) was used to build and develop the PBPK model of bupropion and its metabolites. The model parameters mentioned above were integrated into the PBPK model to simulate PK and DDI. The healthy volunteer population database in the Simcyp simulator is a powerful capability that allows us to assess the combined effects of variations in physiology and pharmacokinetics within populations, as well as formulate variables that are not precise values, but for which distributions of values can be estimated. Each subject is randomly (“Monte Carlo”) generated to have a unique set of generic, anatomic, demographic, and tissue specific parameters, plasma protein binding, hepatic blood flow rate, and pharmacokinetic parameters. The default trial designed by Simcyp is selected to build the model of bupropion and its metabolites. A virtual population of 100 healthy volunteers (10 trials with 10 subjects each) aged 20–50 years with a female/male ratio of 0.5 was used in the simulation of PK following different single oral doses of bupropion (150, 75 and 100 mg).

### 2.6. Simulation of Drug-Drug Interaction (DDI)

In these DDI model, bufuralol, tolterodine, metoprolol, desipramine, and dextromethorphan from the Simcyp simulator library were selected as the CYP2D6 substrates to simulate the DDIs with bupropion and its metabolites. Venlafaxine, whose model was built by us, was also used in the simulation of DDIs. The detailed DDI parameters of bupropion and its metabolites are shown in Table 4.

Trials used in the DDI simulations were designed consistent with the reported clinical studies. The details of the trials were as follows:

(1) The subjects (10 trials × 15 subject, aged 20–50, female/male ratio 0) received 150 mg bupropion or matching placebo orally twice daily for 10 days, and on day 11 the subjects received a single oral dose of 50 mg desipramine. Plasma concentrations of bupropion and desipramine were simulated during the drug treatment period.
(2) The subjects (10 trials × 18 subject, aged 20–50, female/male ratio 0.5) received bupropion (at a daily dose of 150 mg/day) with venlafaxine (at a daily dose of 75 mg/day) for 8 weeks. Plasma concentrations of bupropion and venlafaxine were simulated during the drug treatment period.
(3) The subjects (10 trials × 13 subject, aged 21–64, female/male ratio 0.5) received 150 mg bupropion or matching placebo orally twice daily for 17 days, and on day 18 the subjects received a single oral dose of 30 mg dextromethorphan. Plasma concentrations of bupropion and dextromethorphan were simulated during the drug treatment period.
(4) The subjects (10 trials × 10 subject, aged 20–56, female/male ratio 0.5) received bupropion (at a twice daily dose of 150 mg) with metoprolol (at a twice daily dose of 75 mg) for 12 days. Plasma concentrations of bupropion and metoprolol were simulated during the drug treatment period.
(5) The subjects (10 trials × 10 subject, aged 20–50, female/male ratio 0.5) received 150 mg bupropion or matching placebo orally twice daily for 2 weeks, and on day 15 the subjects received a single oral dose of 20 mg bufuralol or 2 mg tolterodine. Plasma concentrations of bupropion, bufuralol and tolterodine were simulated during the drug treatment period.

The fold-error was used to assess the success of model building and the accuracy of the predicted pharmacokinetic profile and data. Basically, two-fold-error was publicly recognized in the simulation [35,41,42,43,44]. The model was considered to have a goodness-of-fit when the fold-error was less than two. The fold-error was defined as observed/predicted or predicted/observed, where the numerator is greater than the denominator. The DDI effect, expressed as a ratio of AUC and C_max_ in the presence and absence of bupropion, was compared with observed data. The results are listed in Table 5 and Table 6.

### 2.7. PBPK Model for Stereo-Selective Bupropion and Its Metabolites

The PBPK model for stereo-selective bupropion and its metabolites were further developed based on the above model. The corresponding parameters are listed in Table 7. Other parameters not mentioned in Table 7 are similar to those of non-stereo selective bupropion and its metabolites. The absorption and distribution of R-bupropion and S-bupropion were described by the first-order absoption and full PBPK model. For other metabolites, a minimal PBPK model were used to describe this distribution. The in vitro studies showed that R-bupropion was metabolized to form RR-hydroxybupropion via CYP2B6 2C19 and 3A4, respectively, RR-threohydrobupropion and SR-erythrohydrobupropion via carbonyl reductase, and R-4’-hydroxybupropion via CYP2C19; while the S-bupropion was metabolized to form SS-hydroxybupropion via CYP2B6 2C19 and 3A4, respectively, SS-threohydrobupropion and RS-erythrohydrobupropion via carbonyl reductase, and S-4′-hydroxybupropion via CYP2C19 [45]. We have integrated these metabolic pathways into our model. The CYP2J2 was used to define the carbonyl reductase. These corresponding intrinsic clearance rates are calculated by retrograde calculation in Simcyp to account for their proportion in the total clearance rate base on the in vitro study [46]. The total elimination of R-bupropion is divided into 34% hydroxybupropion, 50% threohydrobupropion, 8% erythrohydrobupropion and 8% 4′-hydroxybupropion. For S-bupropion, the proportion of these metabolites are 12% hydroxybupropion, 82% threohydrobupropion, 4% erythrohydrobupropion and 2% 4′-hydroxybupropion, respectively. The V_ss_ of SS-hydroxybupropion and RS-erythrohydrobupropion were predicted with Rogers method and Poulin and Theil method based on the optimized K_p_ value, respectively. The elimination of all metabolites are fitted based on the corresponding observed clinical data.

## 3. Results

### 3.1. Prediction of Bupropion and Its Metabolites Pharmacokinetics

**Figure 2:**
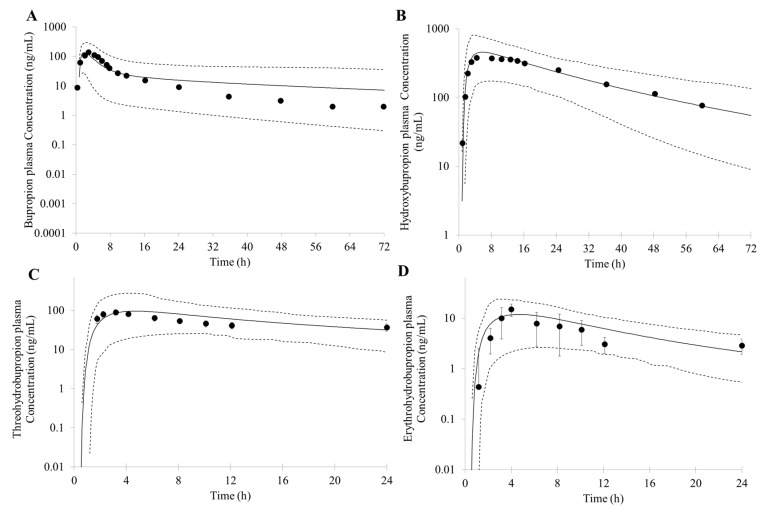
Predicted and observed mean plasma concentration-time profiles of bupropion (**A**); hydroxybupropion (**B**); threohydrobupropion (**C**) and erythrohydrobupropion (**D**) after a single oral dose of 150 mg bupropion. The solid lines represent the predicted mean. The dotted lines represent the 5th and 95th percentile of the predicted values for virtual population. Symbols represent mean observed data (*n* = 17) [34,47].

The PBPK model of bupropion was successfully built based on the parameters in Table 1. The simulated PK profiles after oral doses of 150 mg bupropion are shown in Figure 2. There is a good match between predicted concentration profile and clinically observed data. The predicted C_max_, AUC and T_max_ of bupropion were 136 ng/mL, 1402 ng∙h/mL, and 1.8 h, respectively. All of them were within a two-fold error of the observed results (C_max_ = 143 ng/mL, AUC = 1161 ng∙h/mL and T_max_ = 2.9 h) [47] (Figure 2A).

The simulated concentration-time profiles for hydroxybupropion, threohydrobupropion and erythrohydrobupropion are reasonably well consistent with the observed data based on the model parameters mentioned above (Figure 2B–D). The predicted PK parameters for hydroxybupropion were as follows: C_max_, AUC and T_max_ were 457 ng/mL, 13,564 ng∙h/mL, and 5.8 h, respectively. The observed C_max_, AUC and T_max_ were 433 ng/mL, 16,651 ng∙h/mL, and 7.7 h, respectively [47]. A fold error of less than two was simulated. The predicted C_max_ and AUC for threohydrobupropion were 96 ng/mL and 1358 ng∙h/mL, respectively. The simulated C_max_ and AUC were also in good agreement with (<two-fold error) the observed results (C_max_ = 109 ng/mL, AUC = 1219 ng∙h/mL) [34]. The predicted erythrohydrobupropion C_max_ and AUC were 12 ng/mL and 144 ng∙h/mL, respectively. The simulated C_max_ and AUC were less than 2 fold error compared with the observed results (C_max_ = 15 ng/mL, AUC = 133 ng∙h/mL) [34].

**Figure 3:**
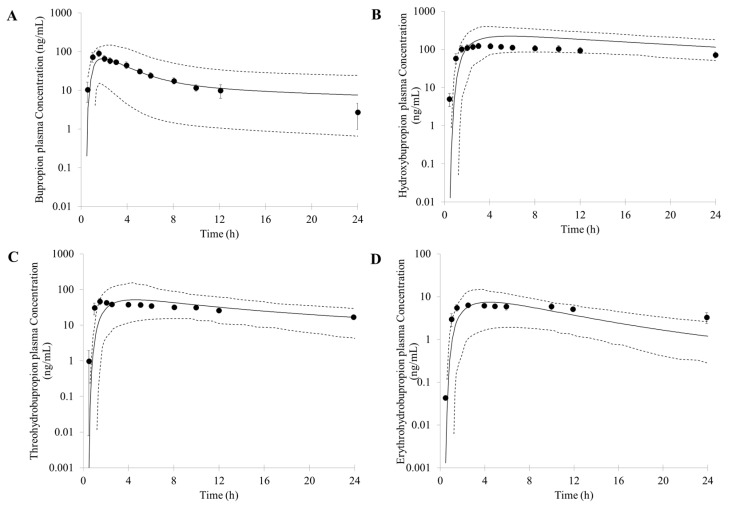
Predicted and observed mean plasma concentration–time profiles of bupropion (**A**); hydroxybupropion (**B**); threohydrobupropion (**C**) and erythrohydrobupropion (**D**) after a single oral dose of 75 mg bupropion. The solid lines represent the predicted mean. The dotted lines represent 5th and 95th percentile of the predicted values for virtual population. Symbols represent mean observed data (*n* = 7) [48].

To verify the PBPK model, the PK profile of bupropion and its metabolites after oral different dose was also simulated and compared with reported data. Following a single oral doses of 75 mg bupropion to healthy subjects, the PK profiles of bupropion and its metabolites are shown in Figure 3. The predicted C_max_ (66 ng/mL), AUC (435 ng∙h/mL) and T_max_ (1.9 h) less than 2 fold error compared with the observed data (C_max_ = 117 ng/mL, AUC = 456 ng∙h/mL and T_max_ = 1.6 h, respectively) (Figure 3A) [48]. For the metabolites, the predicted PK parameters were as follows: C_max_, AUC and T_max_ of hydroxybupropion were 222 ng/mL, 3827 ng∙h/mL, and 5.8 h, respectively; C_max_, AUC and T_max_ of threohydrobupropion were 51 ng/mL, 719 ng∙h/mL, and 4.6 h, respectively; C_max_, AUC and T_max_ of erythrohydrobupropion were 7 ng/mL, 87 ng∙h/mL, and 4.5 h, respectively. The simulated results compared reasonably well with the observed PK data (hydroxybupropion: C_max_ = 134 ng/mL, AUC = 2248 ng∙h/mL, and T_max_ = 4.6 h; threohydrobupropion: C_max_ = 57 ng/mL, AUC = 647 ng∙h/mL, and T_max_ = 1.9 h; erythrohydrobupropion: C_max_ = 7 ng/mL, AUC = 113 ng∙h/mL, and T_max_ = 2.6 h, respectively) (Figure 3B–D) [48]. The simulated results compared reasonably well with the observed data: the predicted PK parameters were within a two-fold error of the observed data, whereas the T_max_ of threohydrobupropion was slightly overpredicted by two-fold error.

**Figure 4:**
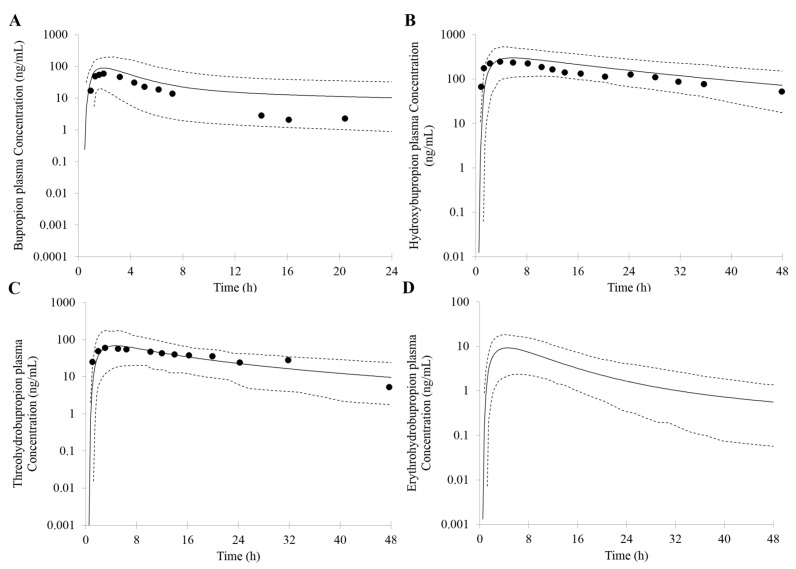
Predicted and observed mean plasma concentration–time profiles of bupropion (**A**); hydroxybupropion (**B**); threohydrobupropion (**C**) and erythrohydrobupropion (**D**) after a single oral dose of 100 mg bupropion. The solid lines represent the predicted mean. The dotted lines represent 5th and 95th percentile of the predicted values for virtual population. Symbols represent mean observed data (*n* = 8) [49].

The PK profiles of bupropion and its metabolites after a single oral dose of 100 mg bupropion are shown in Figure 4. The predicted results were as follows: bupropion: C_max_ = 89 ng/mL, AUC = 586 ng∙h/mL, and T_max_ = 1.9 h; hydroxybupropion: C_max_ = 299 ng/mL, AUC = 7764 ng∙h/mL, and T_max_ = 5.8 h; threohydrobupropion: C_max_ = 68 ng/mL, AUC = 1329 ng∙h/mL, and T_max_ = 4.6 h; erythrohydrobupropion: C_max_ = 9 ng/mL, AUC = 133 ng∙h/mL, and T_max_ = 4.6 h, respectively. They were in agreement with (<two-fold error) the observed PK data (bupropion: C_max_ = 74 ng/mL, AUC = 360 ng∙h/mL, and T_max_ = 1.7 h; hydroxybupropion: C_max_ = 281 ng/mL, AUC = 7468 ng∙h/mL, and T_max_ = 4.2 h; threohydrobupropion: C_max_ = 73 ng/mL, AUC = 1354 ng∙h/mL, and T_max_ = 3.0 h) [49]. However, no concentration-time profile data for erythrohydrobupropio from this study was available for direct comparison.

### 3.2. Prediction of the Bupropion-Desipramine DDI

Desipramine is a substrate of CYP2D6. Although published in vitro data showed that bupropion and a major active metabolite, hydroxybupropion, were relatively weak CYP2D6 inhibitors (IC50 = 58 and 74 μM, respectively) [27], drug interactions resulting in increased exposure of CYP2D6-metabolized drugs following coadministration with bupropion were observed in clinic.

**Figure 5:**
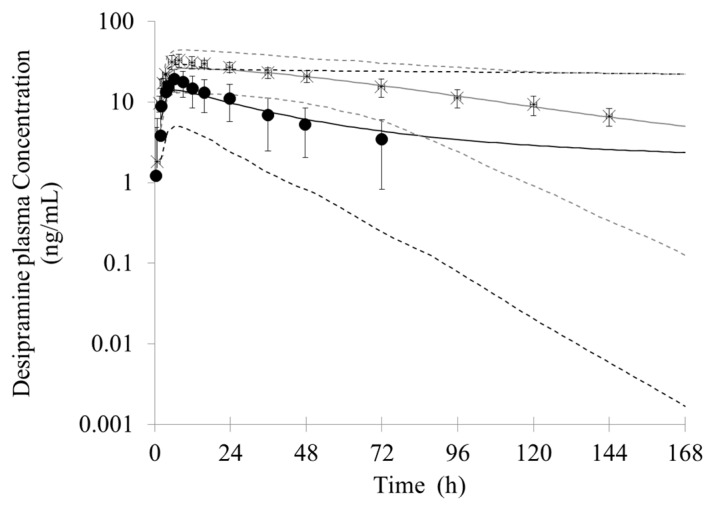
Predicted and observed mean plasma concentration-time profiles of desipramine after a single oral dose of 50 mg desipramine in the absence or presence of a twice-daily dose of 150 mg bupropion. The black solid lines represent the predicted mean concentrations when administered alone; the gray solid lines represent the predicted mean concentrations when co-administered with bupropion. The black and gray dotted lines represent 5th and 95th percentile of the predicted values for virtual population before and after co-administered with bupropion, respectively. Closed circles, observed plasma concentrations when administered alone (*n* = 15) [28]; Stars, observed plasma concentrations when co-administered with bupropion (*n* = 15) [28].

In this simulation, subjects were given a dose of 150 mg bupropion twice a day for 10 days before the administration of a single dose of 50 mg desipramine. The predicted and observed mean plasma concentration–time profiles of desipramine in the absence and presence of bupropion are shown in Figure 5. The predicted and observed pharmacokinetic parameter values are summarized in Table 5. The clinical interaction results showed a 5.2, 1.9 and 2.0-fold increase in the AUC, C_max_ and T_max_ of desipramine, respectively, when desipramine was codosed with bupropion [28]. The simulated results is reasonably well compared to the observed data when all of the inhibition from bupropion and its metabolites were integrated. The predicted AUC, C_max_ and T_max_ ratio were 5.05, 1.79 and 1.84-fold, respectively.

Simultaneously, the contribution of DDI for bupropion and its metabolites were simulated using the PBPK model. The model predicted a 2.27, 1.15 and 1.10-fold increase in desipramine AUC, C_max_ and T_max_, respectively, when bupropion was considered alone as an inhibitor. If each of the metabolites were considered as the only inhibitor, the AUC, C_max_ and T_max_ ratio of metabolites were as follows: hydroxybupropion (4.58, 1.76 and 1.84-fold), threohydrobupropion (3.47, 1.61 and 1.47-fold), and erythrohydrobupropion (2.83, 1.45 and 1.47-fold), respectively. The results indicate that bupropion and its metabolites all are involved in the DDI between bupropion and desipramine. While the inhibition of bupropion is weaker than its metabolites, hydroxybupropion is a relatively strong CYP2D6 inhibitor. 

### 3.3. Prediction of the Bupropion-Venlafaxine DDI

Venlafaxine is another substrate of CYP2D6. The clinical results showed inhibition of venlafaxine metabolism, resulting in a significant, 2.5-fold higher plasma venlafaxine concentration at steady state following co-adminstration of bupropion with venlafaxine [50]. To simulate the DDI, a PBPK model of venlafaxine was developed in the first place. The PBPK model of venlafaxine was successfully built based on the parameters in Table 3. Following a single dose of 75 mg venlafaxine to healthy subjects, the predicted C_max_ (50 ng/mL) and AUC (608 ng∙h/mL) matched the observed data well (C_max_ = 34 ng/mL, AUC = 463 ng∙h/mL) [51].

Then, a simulation of DDI was performed by the PBPK model. In the study, subjects received bupropion (at a daily dose of 150 mg/day) with venlafaxine (at a daily dose of 75 mg/day) for 8 weeks. The simulated results showed a 2.24-fold of C_max_ ratio when the inhibition from bupropion as well as its metabolites were considered. This model can reasonably predict the clinical DDI. The contribution of DDI for bupropion and its metabolites were also analyzed by this model. The predicted C_max_ ratio of bupropion, hydroxybupropion, threohydrobupropion and erythrohydrobupropion were 1.27, 1.94 1.80 and 1.60-fold. The result was similar to the DDI of bupropion on venlafaxine. There was a minimal effect on bupropion, whereas when the inhibition from hydroxybupropion, threohydrobupropion and erythrohydrobupropion were incorporated, significant DDI was captured (Table 5). In general, the inhibition from hydroxybupropion is the strongest, while bupropion has a relatively weak inhibitory effect.

### 3.4. Prediction of DDI between Bupropion with Other Potential CYP2D6 Substrates

The PBPK model was also used to predict more DDI of bupropion on other CYP2D6 substrates. The predicted interaction effect on different drugs was listed in Table 6. A simulation of bupropion inhibits dextromethorphan following a single oral dose of 30 mg dextromethorphan after 17 days of co-administration of bupropion (150 mg twice a day) was performed. According to the model, a 4.06 and 3.05-fold of AUC and C_max_ ratio was predicted, respectively. There are reports showed that interaction occurs when dextromethorphan is co-administered with bupropion in healthy volunteers, the mean dextromethorphan/dextrophan ratio was significantly increased in urine [52]. Even though no concentration-time profile data for the DDI study is available for direct comparison, a significant increase in exposure of dextromethorphan after co-administration of bupropion was predicted by our model.

In a case report, a severe bradycardia occurred between buproprion and metoprolol. It suggested that the serious adverse event might be attributed to the CYP2D6 inhibition of bupropion [53]. Following 12 days multiple oral administration of metoprolol 75 mg twice daily with and without coadministration of bupropion (150 mg twice a day), the predicted AUC and C_max_ ratio of metoprolol were 3.53 and 2.57-fold, respectively. This further confirms the need for caution when combining bupropion with metoprolol.

More drug interactions were studied based on the PBPK model. Following a single oral dose of 20 mg bufuralol or 2 mg tolterodine after 2 weeks of coadministration of bupropion (150 mg twice a day), the predicted AUC and C_max_ ratio of bufuralol were 2.04 and 1.55-fold respectively, and the predicted AUC and C_max_ ratio of tolterodine were 2.91 and 2.17-fold, respectively.

### 3.5. Prediction of Stereo-Selective Bupropion and Its Metabolites Pharmacokinetics and DDI

The above-established PBPK model of stereo-selective bupropion and its metabolites were used to simulate the PK profiles for the subject of 100 mg bupropion administered orally. The results showed that good PK profiles were captured by the PBPK model. All of the predicted C_max_ and AUC were within a two-fold error of the observed results and are shown in Table 8.

On this basis, a DDI between bupropion with desipramine is further simulated following a dose of 150 mg bupropion twice a day for 10 days before the administration of a single dose of 50 mg desipramine. The simulated and observed DDI effect are listed in Table 9. The value of K_i_ was predicted base on IC50 from in vitro reports [55] in the Simcyp model. A 2.53, 1.21, and 1.47-fold of AUC, C_max_ and T_max_ ratio were predicted, respectively, when the inhibition from R-bupropion, RR-hydroxybupropion, threohydrobupropion and erythrohydrobupropion were integrated. Although the predicted DDI was lower than the observed clinical data. The results indicated that the RR-hydroxybupropion was a major coutribution to the inhibition of CYP2D6 from bupropion.

## 4. Discussion

It is common to think that the possibility of causing drug interactions for metabolites compared with the parent drug is low. However, recently, more and more studies have shown that the perpetrator drug’s metabolites may also have a significant impact on CYP-mediated DDI. With the development of the PBPK model, it has been widely used in various stages of drug development, especially in evaluation of DDIs. The PBPK model can simulate a dynamic process which is closer to the in vivo behavior based on in vitro biotransformation and physicochemical parameters. Many studies have successfully evaluated drug interactions using PBPK model [12,56,57,58]. However, only a few studies have built a PBPK model to evaluate DDI caused by inhibitory metabolite [18,59,60,61]. Many compounds, such as bupropion, have an unexpected DDI in clinic, although in vitro study showed that bupropion was a weak CYP2D6 inhibitor. It is possible that the inhibition from metabolites contributes to the observed DDI. To better address this apparent discrepancy between in vitro and in vivo studies, bupropion was chosen as an example, and the PBPK model was employed to describe the complex drug interactions involving inhibitory metabolite.

First, an accurate simulation of PK profiles of both parent and metabolite is required to maximize the confidence in the DDI prediction. Therefore, in our study, many observed PK profiles of different doses were used to verify the bupropion model. A full PBPK distribution model and first order absorption model was used for a good description of bupropion PK profile. Bupropion is mainly metabolized by the liver, and less than 1% of the parent drug is found in the urine [26,29]. In addition to hydroxybupropion that are mediated by CYP enzymes, bupropion is also metabolized by 11β-HSD to form threohydrobupropion and erythrohydrobupropion [62,63]. To better describe and build the PBPK model, CYP2B6 instead of carbonyl reductase was set in Simcyp as the metabolic enzymes of formation of threohydrobupropion and erythrohydrobupropion, and a f_u_,_mic_ was used to correct the expression of carbonyl reductase to obtain the best simulation results compared to observed data.

For those uncertain or unknown parameters, a sensitivity analysis is performed to assess the importance and effect of these parameters in human PK and DDI prediction. In the PBPK model of bupropion, the logP, pKa, and three f_u,mic_ were considered for sensitivity analysis. According to the analysis, the logP, pKa and the f_u,mic_ for formation of erythrohydrobupropion were not sensitive to the prediction of PK. However, f_u,mic_ for formation of hydroxybupropion and threohydrobupropion has a certain impact on the prediction of PK and the f_u,mic_ for formation of erythrohydrobupropion. Thus, the logP and pKa from the drug bank were inputted into the model. The f_u,mic_ for formation of hydroxybupropion and threohydrobupropion were optimized at the 0.16 and 0.003, respectively. The erythrohydrobupropion and threohydrobupropion are formed via reduction of the carbonyl group. Thus, the same f_u,mic_ is integrated into the model. The detailed sensitivity analysis results are shown in Supplementary Figure S1.

Based on the in vitro data and the mechanisms mentioned above, 1% of the fe (fraction of total body clearance via renal excretion) and geometric mean 174 (L/h) of CL were reasonably predicted by the PBPK model. Studies have shown that the CL for bupropion is in the range of 113 to 215 L/h [31,47,49,64,65,66,67,68]. For the PK profile of metabolites, the minimal PBPK distribution model or minimal PBPK distribution model + adjusting compartment distribution model have a good description based on in vitro data, in silico data and clinical PK data. More importantly, the developed PBPK model was well captured the PK profile after oral dose of 75 mg and 100 mg bupropion. 

**Figure 6:**
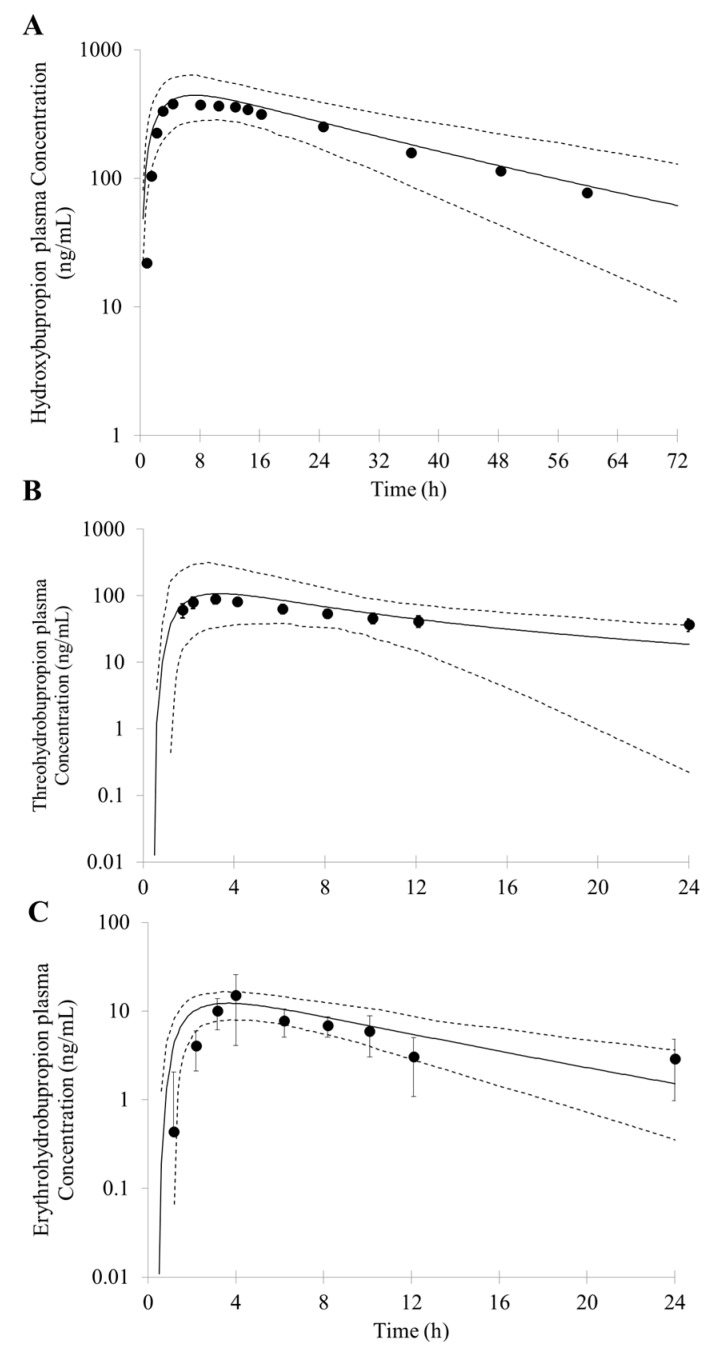
Predicted and observed mean plasma concentration–time profiles of hydroxybupropion with 90 mg (**A**); threohydrobupropion with 30 mg (**B**) and erythrohydrobupropion with 4 mg (**C**). The solid lines represents the predicted mean. The dotted lines represents 5th and 95th percentile of the predicted values for virtual population. Symbols represent mean observed data which is metabolized from a single oral dose of 150 mg bupropion (*n* = 17) [34,47].

In the Simcyp, the user can only simultaneously select one specified inhibitor metabolite to simulate the interaction effects. To better describe the actual clinical DDI, metabolites were regarded as different inhibitors and combined with bupropion to simulate the complex DDI with other CYP2D6 substrates. A sensitivity analysis on the dosage of metabolites was conducted; the results predicted by the model were in good agreement with the observed PK profiles when doses of hydroxybupropion, threohydrobupropion and erythrohydrobupropion were assumed to be 90 mg, 30 mg and 4 mg, respectively. This indicates that the plasma concentration of metabolites formed by single oral 150 mg bupropion is equivalent to plasma levels in vivo after a single oral of 90 mg hydroxybupropion, 30 mg threohydrobupropion and 4 mg erythrohydrobupropion, respectively. (Figure 6). The predicted C_max_ (hydroxybupropion 443 ng/mL threohydrobupropion 107 ng/mL and erythrohydrobupropion 16 ng/mL) and AUC (hydroxybupropion 15,215 ng∙h/mL, threohydrobupropion 1178 ng∙h/mL and erythrohydrobupropion 185 ng∙h/mL) were within 2-fold error of the observed values [34,47].

To sum up, dynamic PK process of bupropion and its metabolites were well characterized in PBPK models. The successful simulations of clinically observed PK profiles build confidence in the prediction and mechanistic understanding of the DDI caused by bupropion, and particularly the unexpected DDI potential contributed by its metabolites. On the basis of the above model, we then applied the PBPK model to predict the clinically observed DDIs involving bupropion and its metabolites as the CYP2D6 inhibitors. The result contribute to the understanding of the involvement and impact of inhibitory metabolites on DDIs observed in the clinic.

In the simulation of bupropion-desipramine interaction, the addition of the inhibitory metabolites into the PBPK model resulted in more accurate prediction of DDIs (AUC and C_max_ ratio) compared with that when only the inhibition of P450 from the parent drug (bupropion) was taken into account. The simulation suggests that bupropion and its metabolites contribute to the DDI between bupropion and desipramin. Although in vitro study showed that the inhibitory potency from strong to weak were erythrohydrobupropion, threohydrobupropion, hydroxybupropion and bupropion, respectively, the simulation of in vivo DDI suggests that hydroxybupropion is the most potent competitive CYP2D6 inhibitor. It can be possible due to the greater exposure of hydroxybupropion. The plasma level of hydroxybupropion is five- to ten-fold higher than the parent drug [29,69,70,71,72]. The exposure of threohydrobupropion is similar to the parent drug; however, it has a stronger in vitro inhibition constant than parent drug and hydroxybupropion. For the erythrobupropion, it is predicted to have similar importance in in vivo DDIs as hydroxybupropion, despite the fact that its plasma concentration is much lower than hydroxybupropion. This may be related to its strongest inhibition constant. Conversely, even though the exposure of bupropion is similar to threohydrobupropion. The PBPK simulation shows bupropion is the weakest competitive CYP2D6 inhibitor. The result may attribute to the relatively weakest inhibition constant.

Consistently, a minimal effect of bupropion on venlafaxine was predicted if only the competitive inhibition from the parent drug was considered. With the addition of the inhibitory metabolites into the PBPK model, there was a more accurate prediction of DDIs. The inhibitory potency from strong to weak was hydroxybupropion, threohydrobupropion, erythrohydrobupropion, and bupropion, respectively.

In the DDI study of bupropion with other CYP2D6 substrates, the significant increase in exposure of dextromethorphan, metoprolol, bufuralol and tolterodine after coadministration of bupropion was predicted. These DDI predictions may explain the occurrence of severe sinus bradycardia after coadministration of bupropion and metoprolol and highlight the need for caution and dosage adjustment when combining bupropion with medications metabolized by CYP2D6.

To better understand the effect of stereo-selective bupropion and its metabolites on the DDI, a stereo-selective PBPK model for bupropion and its metabolites was further developed. The PBPK model considered multiple metabolic pathways including CYP2B6, 2C19, 3A4 and carbonyl reductase, and it is reasonable to describe the proportion of each metabolite in total clearance of bupropion. The simulated PK profile was a good match with the observed clinical data, although, in the simulation of DDI between bupropion with desipramine, the predicted DDI was lower than the observed. The results indicated that RR-hydroxybupropion was a major contributor to the inhibition of CYP2D6 from bupropion. The inhibitory effect of bupropion on CYP2D6 may be the result of synergistic production of all stereo-selective parent drugs and its metabolites. Currently, all inhibitors cannot be simultaneously integrated into the model for simulation. Only four inhibitors can be allowed to integrate into the model in Simcyp. In addition, the stereo-chemical threohydrobupropion and erythrohydrobupropion may have different inhibitory contributions compared to non-stereo-chemical, and the in vitro inhibition rate constants of the stereo-chemical threohydrobupropion and erythrohydrobupropion have not been reported. The PBPK model of stereo-selective bupropion and its metabolites still need to be further improved and optimized after obtaining more data in the future.

Overall, we successfully developed a PBPK model to describe the dynamic PK process of bupropion and its metabolites and understand the involvement and impact of inhibitory metabolites for DDIs observed in the clinic. The present bupropion PBPK model can be useful for predicting inhibition from bupropion in other clinical studies. However, the use of the PBPK model for a true prospective prediction of DDI caused by inhibitory metabolite is still very challenging, as the in vitro inhibition and human PK data for the metabolite are not routinely generated. To maximize confidence in the DDI prediction, more information is needed for the inhibitory potency of the metabolites towards the P450 enzymes.

## Figures and Tables

**Table 1:** Parameters for bupropion used in physiologically-based pharmacokinetic (PBPK) modeling.

Parameter	Bupropion	
	Value	References/Comments
Mol weight (g/mol)	239.74	Drug bank
Log P_o:w_	3.28	Drug bank
pKa	8.22	Drug bank
B/P	0.82	[29]
f_u,p_	0.16	[28]
f_a_	1	[26]
k_a_ (h^−1^)	0.8	[34]
T_lag_ (h)	0.8	[31]
K_p_ scalar	5.4	Simcyp best fit
V_ss_ (L/kg)	19	[31]
Enzyme	CYP2B6	Metabolite: hydroxybupropion
V_max_ (pmol/min per milligram)	3623	[27]
K_m_ (μM)	89	[27]
f_u,mic_	0.16	Assumed = f_u,p_
Enzyme	CYP2B6	Metabolite: threohydrobupropion
V_max_ (pmol/min per milligram)	98.4	[33]
K_m_ (μM)	186.3	[33]
f_u,mic_	0.003	Simcyp best fit, correct expression of carbonyl reductase
Enzyme	CYP2B6	Metabolite: erythrohydrobupropion
V_max_ (pmol/min per milligram)	2.6	[33]
K_m_ (μM)	41.4	[33]
f_u,mic_	0.003	Simcyp best fit, correct expression of carbonyl reductase

B/P, blood-to-plasma ratio; f_u,p_, free fraction in plasma; f_a_, fraction of dose absorbed; k_a_, first-order absorption rate constant; T_lag_, lag time; V_ss_, steady-state volume of distribution; K_m_, Michaelis constant; V_max_, Maximum metabolic rate; f_u,mic_, free fraction in liver microsome.

**Table 2:** Parameters for hydroxybupropion, threohydrobupropion and erythrohydrobupropion used in PBPK modeling.

Parameter	Hydroxybupropion		Threohydrobupropion		Erythrohydrobupropion	
	Value	References/Comments	Value	References/Comments	Value	References/Comments
Mol weight (g/mol)	255.74	ACD-ilab	241.757	ACD-ilab	241.757	ACD-ilab
Log P_o:w_	2.03	ACD-ilab	2.88	ACD-ilab	2.88	ACD-ilab
pKa	7.4	ACD-ilab	7.4	ACD-ilab	9.6	ACD-ilab
B/P	0.82	Assigned using bupropion value	0.82	Assigned using bupropion value	0.82	Assigned using bupropion value
f_u,p_	0.23	[28]	0.58	[28]	0.58	[28]
V_sac_ (L/kg)	0.5	Simcyp best fit	5.83	Simcyp best fit	N/A	
V_ss_ (L/kg)	2.15	Predicted with Rogers method	9.11	Predicted with Rogers method	1.47	Predicted with Rogers method
K_p_ scalar	1	Simcyp default value	1	Simcyp default value	2	Simcyp best fit
CL_po_ (L/h)	5.76	Simcyp best fit	21.15	Simcyp best fit	21.69	Simcyp best fit

B/P, blood-to-plasma ratio; f_u,p_, free fraction in plasma; V_sac_, volume of distribution of compartment; V_ss_, steady-state volume of distribution; CL_po_, oral clearance; N/A, not available. ACD-ilab, the online prediction engine from Advanced Chemistry Development, Inc.

**Table 3:** Parameters for venlafaxine used in PBPK modeling.

Parameter	Venlafaxine	
	Value	References/Comments
Mol weight (g/mol)	277.402	[40]
Log P_o:w_	2.8	[40]
pKa	9.4	[40]
B/P	1.17	[40]
f_u,p_	0.73	[40]
f_a_	0.92	[37]
k_a_ (h^−1^)	1.31	[38]
T_lag_ (h)	1.44	Simcyp best fit
K_p_ scalar	2.3	Predicted with Poulin and Theil method
V_ss_ (L/kg)	7	[38]
Enzyme	CYP2D6	
CLint (µL/min/pmol of isoform)	5.825	Retrograde calculation in Simcyp to account for 80% Hep CL from CYP2D6
CLint-additional (µL/min/mg protein)	11.65	Simcyp predicted

B/P, blood-to-plasma ratio; f_u,p_, free fraction in plasma; f_a_, fraction of dose absorbed; k_a_, first-order absorption rate constant; T_lag_, lag time; V_ss_, steady-state volume of distribution.

**Table 4:** In vitro P450 inhibition parameters for bupropion and its metabolism.

Parameter	Bupropion	Hydroxybupropion	Threohydrobupropion	Erythrohydrobupropion
K_i_ (μM)	21	13	5.4	1.7

All data from [28]. K_i_ here are apparent values, and are corrected for free fraction in microsome (f_u,mic_ = 0.01) estimated in the Simcyp model.

**Table 5:** PBPK model-predicted drug-drug interactions (DDIs) between bupropion and desipramine/venlafaxine.

Inhibitors	AUC Ratio	C_max_ Ratio	T_max_ Ratio
Bupropion + Desipramine (observed)	5.2	1.9	2
Bupropion (predicted)	2.27	1.15	1.10
Hydroxybupropion (predicted)	4.58	1.76	1.84
Threohydrobupropion (predicted)	3.47	1.61	1.47
Erythrohydrobupropion (predicted)	2.83	1.45	1.47
Bup + H-Bup + T-Bup + E-Bup (predicted)	5.05	1.79	1.84
Bupropion + Venlafaxine (observed)	N/A	2.5	N/A
Bupropion (predicted)	1.30	1.27	1
Hydroxybupropion (predicted)	2.49	1.94	1
Threohydrobupropion (predicted)	2.14	1.80	1
Erythrohydrobupropion (predicted)	1.76	1.60	1
Bup + H-Bup + T-Bup + E-Bup (predicted)	3.03	2.24	1

Bup, Bupropion; H-Bup, Hydroxybupropion; T-Bup, Threohydrobupropion; E-Bup, Erythrohydrobupropion; AUC (concentration–time curve) ratio, AUC in the presence of inhibitor/AUC in the absence of inhibitor; C_max_ ratio, C_max_ in the presence of inhibitor/C_max_ in the absence of inhibitor; T_max_ ratio, T_max_ in the presence of inhibitor/T_max_ in the absence of inhibitor; N/A, not available.

**Table 6:** PBPK model-predicted DDIs between bupropion with other potential CYP2D6 substrates.

Substrate	AUC Ratio	C_max_ Ratio
Bufuralol	2.04	1.55
Tolterodine	2.91	2.17
Metoprolol	3.53	2.57
Dextromethorphan	4.06	3.05

AUC ratio, AUC in the presence of inhibitor/AUC in the absence of inhibitor; C_max_ ratio, C_max_ in the presence of inhibitor/C_max_ in the absence of inhibitor.

**Table 7:** Parameters for R-bupropion, S bupropion, RR-hydroxybupropion, SS-hydroxybupropion, RR-threohydrobupropion, SS-threohydrobupropion, SR-erythrohydrobupropion and RS-erythrohydrobupropion used in PBPK modeling.

Parameter	Value	References/Comments	
R-BUP			
Clint (μL/min per pmol)			
CYP2B6	12	Metabolite: RR-OHBUP	Retrograde calculation in Simcyp to account for 34% of total CL [46]
CYP2C19	5.36		
CYP3A4	0.58		
CYP2J2	27	Metabolite: RR-TB	Retrograde calculation in Simcyp to account for 50% of total CL [46]
CYP2J2	4.24	Metabolite: SR-EB	Retrograde calculation in Simcyp to account for 8% of total CL [46]
CYP2C19	4.24	Metabolite: R-4′-OHBUP	Retrograde calculation in Simcyp to account for 8% of total CL [46]
S-BUP			
Clint (μL/min per pmol)			
CYP2B6	20.56	Metabolite: SS-OHBUP	Retrograde calculation in Simcyp to account for 12% of total CL [46]
CYP2C19	12.61		
CYP3A4	1.37		
CYP2J2	236.16	Metabolite: SS-TB	Retrograde calculation in Simcyp to account for 82% of total CL [46]
CYP2J2	11.52	Metabolite: RS-EB	Retrograde calculation in Simcyp to account for 4% of total CL [46]
CYP2C19	5.76	Metabolite: S- 4’-OHBUP	Retrograde calculation in Simcyp to account for 2% of total CL [46]
RR-OHBUP			
CL_po_ (L/h)	6.76	Simcyp best fit	
SS-OHBUP			
V_ss_ (L/kg)	10.5	Predicted with Rogers method	
K_p_ scalar	5	Simcyp best fit	
CL_po_ (L/h)	305.8	Simcyp best fit	
RR-TB			
Vss (L/kg)	4.7	Predicted with Poulin and Theil method	
K_p_ scalar	1	Simcyp default value	
CL_po_ (L/h)	20	Simcyp best fit	
SS-TB			
V_ss_ (L/kg)	4.7	Predicted with Poulin and Theil method	
K_p_ scalar	1	Simcyp default value	
CL_po_ (L/h)	120	Simcyp best fit	
SR-EB			
V_ss_ (L/kg)	3.07	Predicted with Poulin and Theil method	
K_p_ scalar	1	Simcyp default value	
CL_po_ (L/h)	11.69	Simcyp best fit	
RS-EB			
V_ss_ (L/kg)	9.08	Predicted with Poulin and Theil method	
K_p_ scalar	3	Simcyp best fit	
CL_po_ (L/h)	52	Simcyp best fit	

R-BUP, R-Bupropion; S-BUP, S-Bupropion; RR-OHBUP, RR-Hydroxybupropion; SS-OHBUP, SS-Hydroxybupropion; RR-TB, RR-Threohydrobupropion; SS-TB, SS-Threohydrobupropion; SR-EB, SR-Erythrohydrobupropion; RS-EB, RS-Erythrohydrobupropion; R-4′-OHBUP, R-4′-Hydroxybupropion; S-4′-OHBUP, S-4′-Hydroxybupropion.

**Table 8:** Observed versus predicted PK data (AUC, C_max_ and T_max_) of stereo-selective bupropion and its metabolites in the PBPK model of stereo-selective bupropion and its metabolites study.

PK Parameter	AUC (nM·h)		C_max_ (nM)	
	Predicted	Observed [54]	Predicted	Observed [54]
R-BUP	1343.68	1162	196.37	288
S-BUP	291.27	193	53.20	47
RR-OHBUP	37,777.63	37,421	1564.59	1240
SS-OHBUP	524.75	580	33.85	35.9
RR-TB	3228.59	3326	117.19	79.9
SS-TB	1813.4	1433	159.34	168
SR-EB	872.65	942	33.31	30.5
RS-EB	195.48	185	8.12	10.6

R-BUP, R-Bupropion; S-BUP, S-Bupropion; RR-OHBUP, RR-Hydroxybupropion; SS-OHBUP, SS-Hydroxybupropion; RR-TB, RR-Threohydrobupropion; SS-TB, SS-Threohydrobupropion; SR-EB, SR-Erythrohydrobupropion; RS-EB, RS-Erythrohydrobupropion.

**Table 9:** PBPK model-predicted DDIs of between bupropion with desipramine.

Inhibitors	K_i_	AUC Ratio	C_max_ Ratio	T_max_ Ratio
Bupropion + Desipramine (observed)		5.2	1.9	2
R-BUP + RR-OHBUP + EB + TB (predicted)		2.53	1.21	1.47
S-BUP + SS-OHBUP + EB + TB (predicted)		1.93	1.03	1.10
R-BUP (predicted)	12.5	1.83	0.96	1.10
S-BUP (predicted)	0.91	1.84	0.97	1.10
RR-OHBUP (predicted)	1.5	2.45	1.19	1.47
SS-OHBUP (predicted)	4.3	1.84	0.97	1.10
Threohydrobupropion (predicted)	3.97	1.88	0.99	1.10
Erythrohydrobupropion (predicted)	0.91	1.87	0.98	1.10

Bup, Bupropion; H-Bup, Hydroxybupropion; T-Bup, Threohydrobupropion; E-Bup, Erythrohydrobupropion; AUC ratio, AUC in the presence of inhibitor/AUC in the absence of inhibitor; C_max_ ratio, C_max_ in the presence of inhibitor/C_max_ in the absence of inhibitor; T_max_ ratio, T_max_ in the presence of inhibitor/T_max_ in the absence of inhibitor.

## Supplementary Materials

The following are available online at www.mdpi.com/1999-4923/10/1/1/s1, Figure S1: Sensitivity analysis results expressed as plasma concentration-time profiles from Simcyp PBPK model simulations: (A) logP; (B) pKa; (C) f_u,mic_ for formation of hydroxybupropion, (D) f_u,mic_ for formation of threohydrobupropion, (E) f_u,mic_ for formation of erythrohydrobupropion. The parameter ranges assessed were logP, 0.33−32.8, pKa, 0.82−14.00, f_u,mic_, 0.0005−1.

Supplementary File 1
