# Supplementary material for: Prediction of Drug-Drug Interactions with Bupropion and Its Metabolites as CYP2D6 Inhibitors Using a Physiologically-Based Pharmacokinetic Model
Source: Pharmaceutics. 2017 Dec 21;10(1):1. doi: 10.3390/pharmaceutics10010001 (PMC5874814; doi:10.3390/pharmaceutics10010001)
Supplement: Supplementary File 1 [file pharmaceutics-10-00001-s001.pdf]

Supplementary Materials: Prediction of Drug-Drug Interactions with Bupropion and Its Metabolites as CYP2D6 Inhibitors Using a Physiologically-Based Pharmacokinetic Model

Caifu Xue, Xunjie Zhang and Weimin Cai *

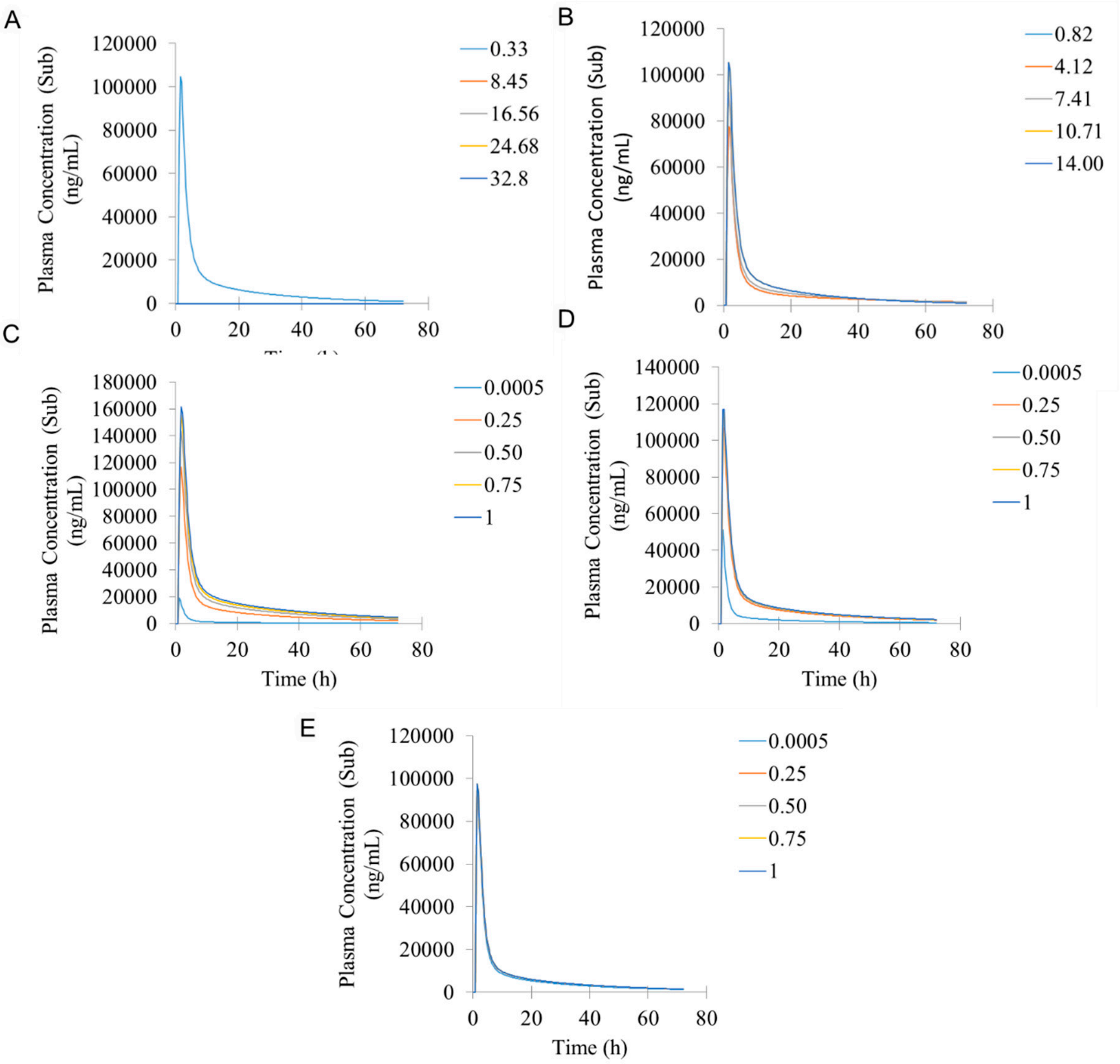

Figure S1. Sensitivity analysis results expressed as plasma concentration-time profiles from Simcyp PBPK model simulations: (**A**) logP; (**B**) pKa; (**C**) $f_{u,mic}$ for formation of hydroxybupropion, (**D**) $f_{u,mic}$ for formation of threohydrobupropion, (**E**) $f_{u,mic}$ for formation of erythrohydrobupropion. The parameter ranges assessed were logP, 0.33−32.8, pKa, 0.82−14.00, $f_{u,mic}$, 0.0005−1.
